# Community Profiling of Seed Endophytes from the Pb-Zn Hyperaccumulator *Noccaea caerulescens* and Their Plant Growth Promotion Potential

**DOI:** 10.3390/plants12030643

**Published:** 2023-02-01

**Authors:** Tori Langill, Lambert-Paul Jorissen, Ewa Oleńska, Małgorzata Wójcik, Jaco Vangronsveld, Sofie Thijs

**Affiliations:** 1 Environmental Biology, Centre for Environmental Sciences, Hasselt University, Agoralaan Building D, 3590 Diepenbeek, Belgium; 2 Faculty of Biology, University of Bialystok, 1J Ciołkowski, 15-245 Bialystok, Poland; 3 Department of Plant Physiology and Biophysics, Institute of Biological Sciences, Maria Curie-Skłodowska University, 20-033 Lublin, Poland

**Keywords:** community profiling, growth promotion, *Noccaea caerulescens*, Pb-Zn hyperaccumulator, seed endophytes

## Abstract

Endophytes within plants are known to be crucial for plant fitness, and while their presence and functions in many compartments have been studied in depth, the research on seed endophytes is still limited. This work aimed to characterize the seed endophytic and rhizospheric bacterial community of two *Noccaea caerulescens* Pb-Zn hyperaccumulator populations, growing on two heavy-metal-polluted sites in Belgium. Cultured representatives were evaluated for their potential to enhance seed germination and root length of the model species *Arabidopsis thaliana*. The results indicated that the community structure within the seed is conserved between the two locations, comprising mainly of Proteobacteria (seeds), and Actinobacteria in the bulk soil. Root length of *A. thaliana* was significantly increased when inoculated with *Sphingomonas vulcanisoli*. The results of this paper offer insights into the importance of the selection of the core seed endophytic microbiome and highlight the precarious symbiotic relationship they have with the plant and seed.

## 1. Introduction

Plants have co-evolved with diverse microbial communities [1]. Amongst them, the seed endophytes are the first microbes to colonize a successful plant [2], and given the fact that they can be transmitted vertically, it seems reasonable that seed endophytes actually hold the key to successful germination in marginal conditions [3].

**Figure 1:**
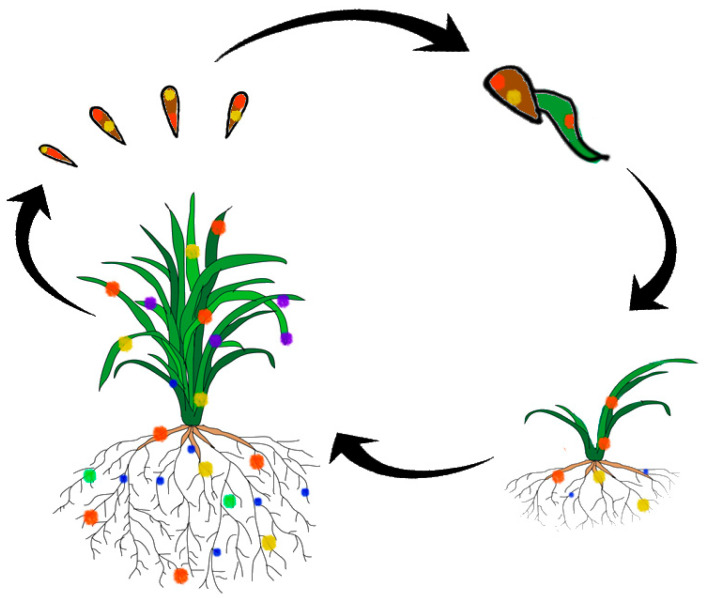
Seeds from the mature plant contain specific microbes which are essential for successful germination. These seed endophytes become the first microbes to inhabit the new plant, as it establishes itself in its environment. Each color represents a phylum of endophytes, and highlights how not all endophytes present in the mature plant become part of the seed.

Numerous bacterial species have been identified from a wide array of different plant seeds [4]. More precisely, they can be found in the seed embryo, seed coat and storage tissues (endosperm and/or perisperm) of the seed [5]. Seed-borne microbes are believed to be transmitted through xylem tissues, through the stigma of the maternal plant, or recruited from the external environment [6]. These bacterial communities live in a mutualistic relationship with their plant host. The seed interior provides the bacteria with a protective sanctuary, where they can grow and reproduce without disturbing the life cycle of their host (Figure 1).

Additionally, it has been verified that seed-endophytes play major roles in plant-growth promotion [7]. Through the production of growth hormones and enzymes such as indole-3-acetic acid (IAA) and 1-aminocyclopropane-1-carboxylate (ACC) deaminase, root growth is promoted by stimulating cell elongation [8]. It has also been shown that the seed microbiome plays an essential role in the improvement of nutrient uptake by the plant, nitrogen fixation, phosphate solubilization, seed germination and protecting the plant against abiotic and biotic stresses [9]. As a result of the positive effects the endophytes provide on plant growth, interest in the application of endophytic bacteria has grown in the agricultural sector, in particular for germination of crop seeds on marginal land.

Seeds endophytes from hyperaccumulator *Noccaea caerulescens* are of particular interest, given the plant not only germinates on, but thrives on zinc-polluted soil [7]. The samples used in this research originate from the former mine site in Plombières, Belgium, which is polluted with heavy metals resulting from historical mining activities—most notably, lead, cadmium, and zinc pollution [10]. Metal hyperaccumulating plants have the notable property of being able to tolerate toxic levels of heavy metals such as cadmium (Cd), zinc (Zn) and nickel (Ni). Certain ecotypes of *N. caerulescens* can accumulate up to 30,000 ppm of Zn and approximately 10,000 ppm of Cd in their shoot biomass, whilst these concentrations would be highly toxic for most terrestrial plants [11]. Hence, *N. caerulescens*, and more specifically, the seed-endophytes associated with *N. caerulescens*, provide an excellent model system to study the mechanisms of heavy metal hyperaccumulation and the adaptation of terrestrial plants to heavy-metal-polluted soils. It is hypothesized that metal accumulators use stored metals as a defense mechanism against pests and pathogens [12]. Previous findings support the idea of local adaptation of endophyte communities in natural populations of *N. caerulescens* and provide evidence for the theory of pathogens being a selective force in the evolution of metal hyperaccumulation [13]. The transmission and colonization of bacteria with beneficial traits for the host indicates that seed-endophyte communities are selected and passed on to successive generations [14]. The interaction between seed microbiota and the host plant may therefore have been a key factor in the adaptation for survival and reproduction in these hostile environments.

Because these plants, and by proxy, their seed endophytes, are so unique, understanding the community structure of the microbes, as well as determining how well culturable microbes from the seeds can affect different plants when applied to their seeds, is of immense value. This paper addresses the community structure of the endophytic community from *N. caerulescens* through Illumina MiSeq sequencing, comparing samples taken from two distantly located sites in Belgium. Both sample sites are polluted with Cd, Zn, and Pb (Overpelt and Plombières). Bacterial isolates were taken from *N. caerulescens* seeds and applied to *Arabidopsis thaliana* seeds, in order to see if the effect of introducing new seed endophytes had an impact on germination, root length and plant size. This research aims to provide the foundation for a novel method of seed treatment whereby plants could be given assistance in germinating in less than ideal conditions due to pollution. It also looks at the relationship between the seed endophytic community and growth location of the plant.

It is hypothesized that introducing seed endophytes from *N. caerulescens* to the seeds of *A. thaliana* will improve the germination and overall plant health and biomass when *A. thaliana* is forced to germinate in marginal conditions. It is also hypothesized that the seed microbiome reflected from the Illumina sequencing will be similar in all seeds, regardless of the location they originated from, adding weight to the working theory that seed endophytes are conserved generationally and are therefore incredibly important to plant health and germination success.

## 2. Results

### 2.1. Culturable Bacterial Identification through Sequencing and Alignment

The resulting raw sequence data was assigned to a taxonomic classification by alignment using the nucleotide Basic Local Alignment Search Tool (BLASTn) against the nucleotide database (NCBI database) [15].

The BLASTn results (Table 1) from the raw sequence data input showed a high identity coverage (>92%) with matching bacterial strains found in the NCBI database. Except for isolate number 11, which had a noteworthy match with an uncultured strain of the class Alphaproteobacteria, all isolates were classified to the level of genus. Isolate numbers 1 and 6 matched with a specific bacterial strain (*Mucilaginibacter* sp. 104 and *Sphingomonas* sp. AP4-R1). The taxonomic classification (Table 2) of the cultivated endophytic bacteria was generated by the NCBI taxonomy browser and subdivided in ascending order of Phylum, Class, Order and Genus [16]. A total of 64% of the cultivated *N. caerulescens* seed isolates belong to the bacterial class Alphaproteobacteria, making it the predominant class isolated from the seeds. The remaining isolates belong to 3 Betaproteobacteria, Sphingobacteriia and Bacilli.

**Figure 2:**
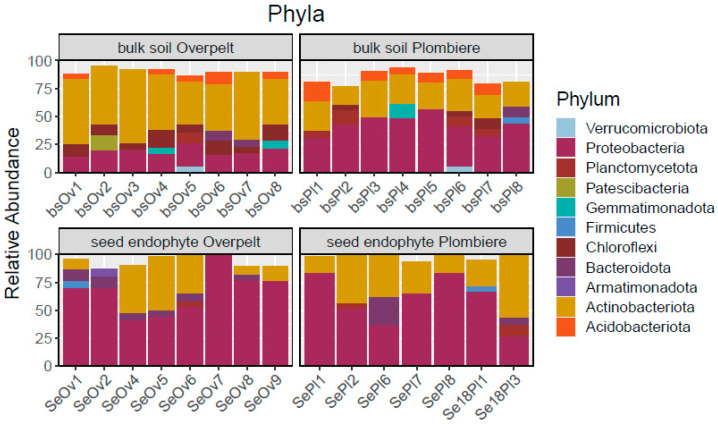
Phyla community results of *N. caerulescens* from two locations—Overpelt and Plombières, Belgium—separated into compartments addressing the bulk soil and the seed endophytes.

**Figure 3:**
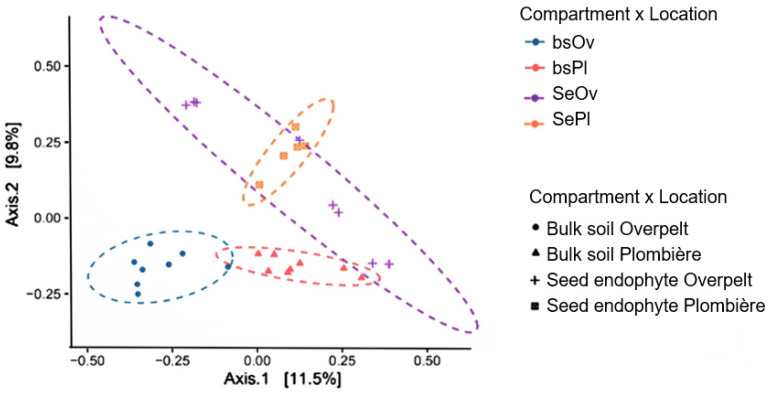
NMDS analysis with Bray–Curtis distance of bulk soil and seed endophytic communities from Overpelt and Plombières, Belgium.

The highest instance of Actinobacteria and the lowest instance of Proteobacteria were found in the bulk soil collected from Overpelt, Belgium. Gemmatimonadota was found in both locations in the bulk soil, but interestingly it was not present in the seed. The same is true of Acidobacteriota. The seed predominantly hosts Proteobacteria (Figure 2). Bulk soil bacterial communities group separately, while the seed communities’ group closer together in regard to NMDS analysis (Figure 3).

**Figure 4:**
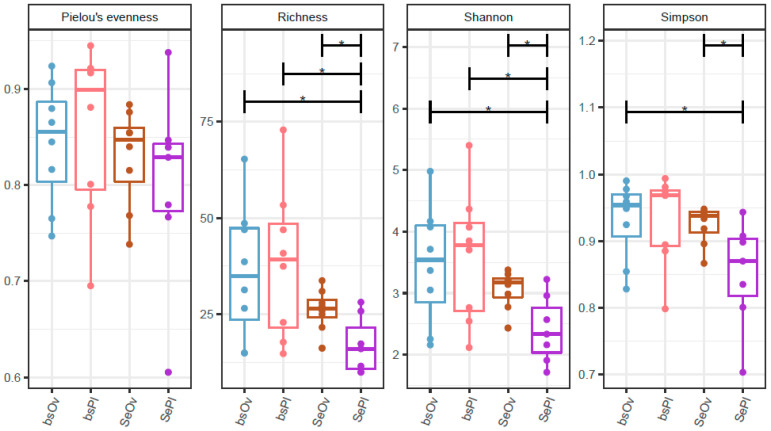
The species richness and diversity indexes of the bulk soil and seed endophytes of *N. caerulescens* from Overpelt and Plombières, Belgium. (* *p* < 0.05, ANOVA).

Species richness between the two sampled sites indicates similar diversity at both locations. Species richness in the seeds from Plombières shows a more conserved range of bacteria. The Shannon–Wiener index show significant differences between the microbe life present within the bulk soil when compared to the more selective seed endophytes present at Plombières (Figure 4).

### 2.2. Seed Germination and Primary Root Growth with Bacterial Inoculation

**Figure 5:**
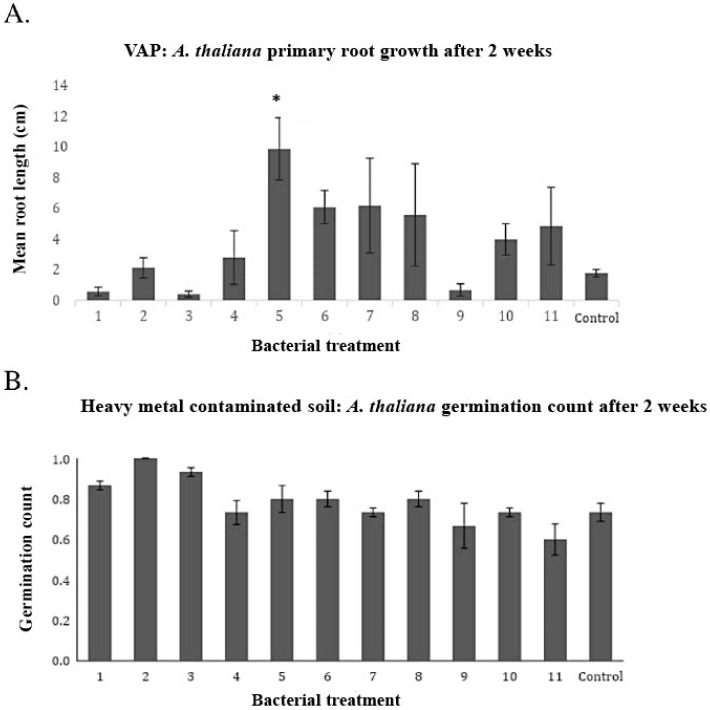
(**A**) Primary root growth of A. thaliana seedlings treated with bacteria isolated from seeds of *Noccaea caerulescens*. The seeds were sown on Vertical Agar Plates (VAPs) with 20× basal medium and primary root growth was measured after 2 weeks. (**B**) Germination count of *A. thaliana* seeds treated with bacteria isolated from seeds of *N. caerulescens*. The seeds were sown on pots with heavy-metal-contaminated soil (Plombières, BE), and germination was counted after 2 weeks. A significant difference was analyzed by a one-way ANOVA Tukey HSD test and a significance level of *p* < 0.05 is indicated by an asterisk. All bacterial treatment numbers found on the *x*-axis correspond with Table 1.

Root growth after 2 weeks showed higher on average root length, and this was statistically significant (*p* < 0.05) in the case of treatment with Sphingomonas vulcanisoli (sample 5). No statistical differences were seen in germination, but there was an observed effect indicating better germination with seed treatment (Figure 5).

### 2.3. A. thaliana Response in the Presence of High Heavy Metal Concentrations and Bacterial Inoculation

**Figure 6:**
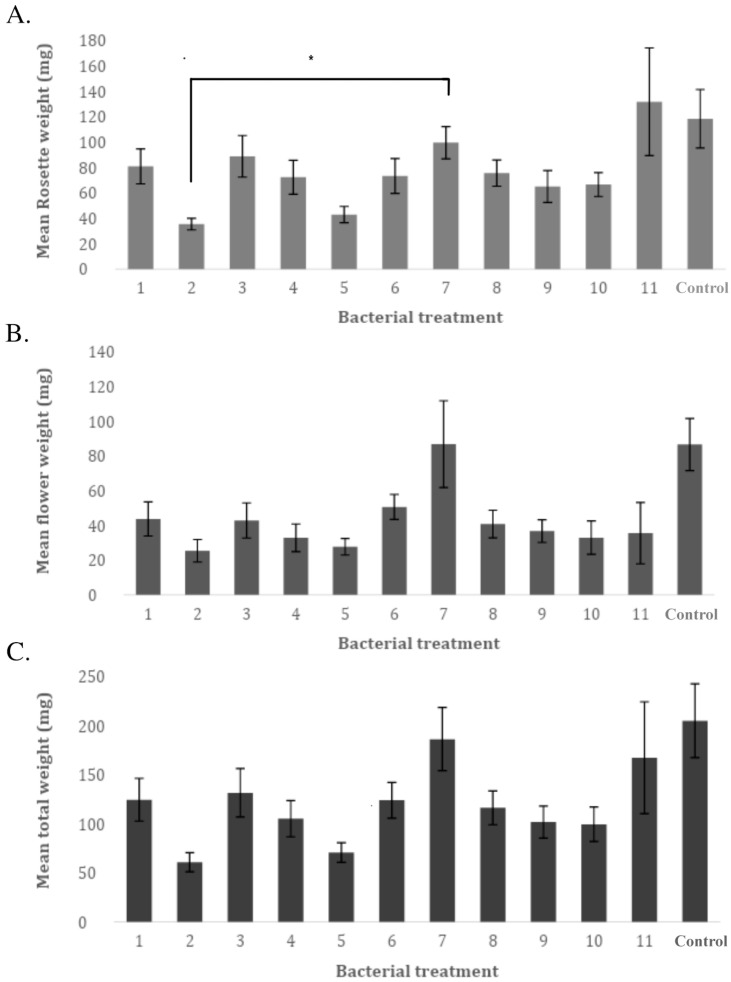
(**A**) Mean Rosette weight. (**B**) Mean flower weight. (**C**) Mean Total weight (root weight not included) of *A. thaliana* growing on heavy-metal-polluted soil pots. All *A. thaliana* seeds were inoculated with *N. caerulescens* seed isolates except for number 12 (no treatment: control). Weight was measured after 3 weeks of growth. A significant difference in biomass was analyzed by a one-way ANOVA Tukey HSD test and significance of *p* < 0.05 is indicated by an asterisk. All bacterial treatment numbers found on the *x*-axis correspond with Table 1.

For the weights (rosette, flower, and total) of treated *A. thaliana* plants, no significant improvement was found when compared to the control. Notably, the total weight of plants (Figure 6C) inoculated with isolate number 7 showed higher total biomass compared to other treatments. This observation can be followed by comparing treatments to each other through a one-way ANOVA Tukey HSD. A significant difference is found for treatment 2 (*p* < 0.05) when compared to treatment 7. For the weight of the *A. thaliana* flower (Figure 6B), similar observations as for the total weight can be made with treatment 7 resulting in a higher flower weight than some other treatments. Notably, this is the one instance in which treatment 7 offers higher biomass than the control. For the rosette weight (Figure 6A), the difference in weight from treatment 7 compared to other treatments is not as remarkable as with the flower and total weight. More noticeable is the average rosette weight of *A. thaliana* (131.86 mg) treated with isolate number 11 (Alpha proteobacteria bacterium (uncultured)).

## 3. Discussion

### 3.1. Bacterial Isolate Affect upon A. thaliana Seeds

The isolation of seed endophytic bacteria from the *N. caerulescens* seeds combined with the Macrogen sequencing data and Illumina MiSeq sequencing delivered some interesting results. Eleven different isolates (Table 1 and Table 2) from the seeds were cultivated on 1/10 enriched medium and consisted mainly of the bacterial class α-Proteobacteria (~64%). When the sequencing results in this study are compared to other studies focusing on the *N. caerulescens* seed microbiome, some similarities can be found. Durand et al. (2021) studied the bacterial seed community of *N. caerulescens* across 14 sites in France [7]. However, the data collected from the study of Durand et al. (2021) did not include a bacterial cultivation method, and bacterial characterization was performed only through Illumina MiSeq high throughput 16S rRNA gene sequencing. Our research and findings further solidify their results in regard to the core microbiome of the community structure of *N. caerulescens* seeds, as well as addressing the differences to the community in regard to bulk soil. It is also important to mention that the relative abundance of the phyla is correlated to environmental factors (pH, soil elements and altitude). Lastly, the host plants’ unique genetics plays a significant role in the level of seed endophytic community diversity [7]. Their metabarcoding revealed a large seed core microbiome mainly composed of two bacterial classes: ɣ-Proteobacteria (56.56%) and α-Proteobacteria (32.23%). Whilst other bacterial classes did not contribute culturable results in our study (ɣ-Proteobacteria and Actinobacteria), similarity in the structure is found with α-Proteobacteria, Bacteroidetes and Firmicutes.

An interesting observation is the high abundance of Sphingomonas strains (28.46%) in their study, making it the most abundant group of bacteria from the α-Proteobacteria class. This is reflected in our culturable results with Sphingomonas strains being the most abundant group among the isolates (36.36%). Although our data reflects a high similarity in bacterial seed community composition with the *N. caerulescens* samples from France, distinction in methods for collecting data should be taken into account, as well as the knowledge that culturable results from a single growth medium will never truly accurately reflect community sequencing results due to the different growth preferences of individual bacteria [17].

As Eevers et al. (2015) already stated, it is hypothesized that only 0.001–1% of all plant-associated bacteria are cultivable [18]. Thus, it is expected that the cultivation of *N. caerulescens* isolated seed bacteria on 1/10 enriched media resulted in a specific selection of bacteria, and this is not a reflection of the true diversity and levels of phyla present within the seed. Sequencing results confirm this, showing different levels of phyla compared to what was able to be cultivated (Figure 1 and Table 2).

Treatment 5, identified as *Sphingomonas vulcanisoli* through 16S sequencing, showed itself to be a culturable candidate of interest as it is the only treatment to show positive significant results on the root length of *A. thaliana* (Figure 5). It also showed similar germination to the control, indicating that the addition of this bacteria is not harmful to the seed or plant. This is important to note as seeds of plants tend to often be colonized with deleterious rhizobacteria (DRB), which can negatively affect germination and actually suppress plant growth [19]. DRB affect plant growth through bacterial allelochemicals, and they tend to associate primarily with weed plant species [20,21,22]. *N. caerulescens* and *A. thaliana* both fall into the weed category, meaning both seeds could potentially play host to, and be affected by, DRB. Therefore, it is worth mentioning that perhaps the lack of significance found in our PGP results, which is due to the allelopathic effect of the potential DRB specifically on *A. thaliana* and another test subject, which is not a weed, potentially could have positive and significant results.

*Sphingomonas* has previously been highlighted to be a key endophyte within the seed of *A. thaliana* [23]. This could be a contributing factor as to why this bacterium performed better than the others tested, since it was not foreign to the seed; rather, only the level of exposure was increased. It is unlikely that *S. vulcanisoli* is a DRB, given that the majority of DRB are *Pseudomonas* or Firmicutes [19].

Notably, we observed three treatments which caused detriment to the root length, and although this detriment was not significant, it is worthy of note. Treatments 1, 3, and 9 (*Mucilaginibacter* sp. 1042, *Mucilaginibacter* sp. and *Paenibacillus* sp., respectively), appeared to cause impairment of root development. The two working theories for this negative effect on growth are as follows: the bacteria could be DRB, which would inhibit plant growth in a non-significant way, or this is due to these microbes being foreign to the seed endophytic community within *A. thaliana*, which may have caused unnecessary stress to the seed, hindering root development upon sprouting [24]. Of course, further research is needed to either confirm or deny these theories.

This detrimental trend is reflected further in our results, upon looking at total biomass, as the aforementioned treatments offered smaller plants in all compartments (Figure 6). Only *Sphingomonas wittichii* showed promise in increasing biomass, although it was not significant, and it increased flower weight. Overall biomass was on par with the control. This bacterium also showed potential since this treatment resulted in greater root length when compared to the control and did not hinder germination. Given its predominant status as a well-known degrader of environmental contaminants, it should be given high priority and greatly considered for use in industry [25].

Still, as the rest of the isolates did not perform as hoped upon the *A. thaliana* seeds, it stands to reason they could be better tested on a more resilient seed, such as *mais* or *Medicago sativa,* as both species are crops, not weeds. Treatment involving more than a single isolate should also be considered, because as we delve into community structure, we can see that seeds are made up of a delicate equilibrium, not a single predominant entity.

### 3.2. The Core Microbiome of N. caerulescens

Within the seed of *N. caerulescens* we see an abundance of proteobacteria and actinobacteria (Figure 2). In fact, these two phyla make up almost the entire endophytic community. This corresponds with the results of Durand et al. (2021), who noted that the seed endophytic community across 14 sites in France also had predominantly proteobacteria [7]. This is again supported by our previously discussed cultured, which were predominantly proteobacteria (Table 2). Interestingly, we can see that the bulk soil communities from the separate sites are different enough to group separately when an NMDS analysis is performed, while the seeds appear more similar in community (Figure 3). This strengthens the hypothesis that the seed communities are conserved through generations, and regardless of environment, seeds of the same species should host a similar if not identical core community of seed endophytes. Addressing the bulk soil community, there is a solid presence of Acidobacteriota and Gemmatimonadota, yet neither of these phyla are present within the seed, except for one replicate from Overpelt, which showed a small amount of Acidobacteria.

This result was not repeated in any of the other replicates and can therefore be classified as an outlier. Gemmatimonadota is known to be difficult to culture, with only six culturable strains found to date [26]. It is also noted to commonly be found in the rhizosphere of plants, indicating it may offer benefit to the plant, particularly the root of hyperaccumulators [26]. However, it is unlikely that they play a role in seed germination or protection, as they do not occur in the seed. We strongly believe that the core microbiome found in the seed is integral to the successful germination of the plant [27]. The most present class of microbiota present in the seeds from both sites was gammaproteobacterial. Contrastingly, the most prevalent class found in the bulk soil at Overpelt was Actinobacteria, and the most prevalent class found in the bulk soil at Plombières was Alphaproteobacteria. This is interesting because it highlights how the seed communities are similar, despite the two separate sites being distantly located and having statistical differences in the bulk soil. Both sites, though similar in pollution, host a plethora of different classes of microbiotic life within the bulk soil, and yet, the seed microbiome is conserved. The NMDS analysis (Figure 3) supports this as it is clear to see the two populations are separate from each other. Further study on the dynamics of the bacteria present within the seed should be considered to assist better in seed protection against frost or drought, as well as to optimize the likelihood of germination in general.

In Figure 4, we see that there is a significant difference between the seed communities from Plombières and Overpelt in regard to the Simpson’s diversity index, the Shannon–Wiener index, and species richness. This is not what we would expect to see since we hypothesize that the seed core microbiome is conserved. However, it is important that this data not be looked at without the beta analysis (Figure 3), in order to provide the complete picture. It is therefore believed, based on the statistical findings of our study, that there is significant diversity found between each seed, but not between each population of seeds. Further experiments must be performed including an increased sample size so that further support can be given to this theory.

## 4. Materials and Methods

### 4.1. Isolation and Cultivation of N. caerulescens Seed Endophytes

Seeds were harvested from *N. caerulescens* at a heavy-metal-polluted site in Plombières, Belgium (50.7379° N, 5.9611° E). The seed surface was sterilized by using a 1% sodium hypochlorite (NaClO) solution [4]. The seeds were rinsed 5 times with sterile distilled water (dH2O). The sterilized seed coat was crushed with an ethanol-disinfected mortar and pestle, whereby dH2O was added to make a solution of the resulting seed components. A ten-fold serial dilution of unquantified seed material was made. The 10^−4^, 10^−5^, and 10^−6^ concentrations of the dilution series were selected for further usage. Bacteria were grown on 1/10 diluted enriched media (0.070 g CaCl_2_·2H_2_O, 0.200 g D-(+)-glucose·H_2_O, 1 g NaCl, 2 g Tryptone, 1 g Yeast Extract, 15 g Agar) [28]. An amount of 100 µL for every dilution concentration above was applied to separate triplicated enriched media Petri dishes. The plates were incubated for 10 days, at 27.5 °C. The plate with the 10^−4^ dilution had the best visible yield and was further used for the isolation of pure colonies. Unique bacteria were selected based on the morphology and color of their colonies. In total, 11 different bacterial colonies were collected, and each was purified on solid 1/10 enriched medium. The bacteria were incubated for 10 days, at 27.5 °C.

### 4.2. DNA Extraction, Sequencing, and Identification

The pure colonies were collected and suspended in 200 µL Phosphate-Buffered Saline (PBS) solution [29]. For every individual colony, 3 replicates and a negative control without bacterial suspension were made and resulted in a total of 33 solutions of individual bacterial colonies. DNeasy^®^ 96 Blood and Tissue Kit (Qiagen) was used to produce purified bacterial DNA solutions for every sample. For the resulting DNA solutions, the Q5 ^®^ Hot Start High-Fidelity DNA Polymerase (M0493) kit was used. The forward primer 27F (5′AGA GTT TGA TCC TGG CTC AG-3′) and reverse primer 1492R (5′-GTT TAC CTT GTT ACG ACT T-3′) amplified the 16S rRNA gene. Samples were confirmed on an agarose gel (100 mL 1× TAE buffer, 1.5 g agarose, 10 µL gelred). The amplified DNA samples were sent to Macrogen Europe (Amsterdam, The Netherlands) for 16S rDNA gene Sanger sequencing. The resulting raw sequence data was assigned to a taxonomic classification by alignment using the nucleotide Basic Local Alignment Search Tool (BLASTn) with the nucleotide database of the National Center for Biotechnology Information (NCBI) [15].

### 4.3. Vertical Agar Plates (VAP) Primary Root Measurement and Seed Germination Count

The medium for the VAPs consists of: 0.5 mM KNO_3_, 0.02 mM MgSO_4_·7H_2_O, 0.02 mM CaCl_2_·6H_2_O, 0.022 mM NaH_2_PO_4_, 0.94 μM MnSO_4_·H_2_O, 0.02 mM (NH_4_)2SO_4_ (macronutrients), 90 nM KI, 0.97 nM H_3_BO_3_, 0.14 nM ZnSO4·7H2O, 2 nM CuSO_4_·5H_2_O, 20.6 nM Na_2_MoO_4_·2H_2_O, 2.6 nM CoSO_4_·H_2_O (micronutrients), 3.6 μM FeCl_3_, 2.56 mM 2-[N-Morpholino] ethanesulfonic acid MES (Sigma), pH = 5.7 at a 50× dilution. The isolated bacteria were grown on 1/10 enriched media for 1 week, at 30 °C, with an agitation of 100 rpm. They were centrifuged at 3000 rpm for 10 min and washed afterwards with PBS buffer before being resuspended in sterile dH_2_O. The *A. thaliana* seed surface was sterilized for 1 min in 70% ethanol and resuspended afterwards in dH_2_O [30]. Following this, the seeds were immersed in the bacterial suspension solutions, where they were incubated for 3 days, at 5 °C. A negative control without bacterial treatment was included. After the incubation, 10 seeds from *A. thaliana* of each specific bacterial treatment were transferred to individual VAPs, respectively. The VAPs were incubated vertically in a growth chamber with UV lighting. Primary root growth was monitored every 2 days. After 2 weeks, the plates were scanned for 2D pictures, and root growth was measured manually. The seed germination was observed and counted every 2 days.

### 4.4. A. thaliana Response in the Presence of Heavy Metal Contamination and Bacterial Inoculation

Isolated bacteria were grown in enriched broth for 1 week, at 30 °C, with an agitation of 100 rpm. They were centrifuged at 3000 rpm for 10 min and washed afterwards with a PBS buffer, before being resuspended in 50 mL sterile dH20. The Arabidopsis thaliana seed surface was sterilized for 1 min in 70% ethanol. The seeds were immersed in the bacterial supernatant solutions, where they incubated for 3 days, at 5 °C. *A. thaliana* seeds were sown on heavy-metal-polluted soil samples (Table 3) from Overpelt, Belgium where Cd pollution is high due to historic Zinc smelting in the area. This soil was chosen as more bioavailable heavy metals were present when compared to the soil of Plombières, whereby we could better observe the stress effect on the plants. A total of 15 seeds were sown in triplicate in separate soil pots. The plants were grown under greenhouse conditions (with 170 mol m^−2^ s^−1^ PAR, 22 °C/18 °C degrees, 12/12 day–night cycle and 65% RH) and germination was observed every 2 days. After 3 weeks of growth, the plants were harvested and collected for weight measurement. For every bacterial treatment, the weight of the rosette and flowers (stem + generative parts) were measured separately. For total plant weight, the roots were not included.

### 4.5. Endophytic Colony and Bulk Soil Colony Extraction for NGS

The aforementioned serial dilution of seed material was used for DNA extraction using the EchoLUTION Plant DNA Kit (Bio ECHO). During harvesting of seeds at Plombières and Overpelt, bulk soil was collected. DNA was extracted from soil samples using the MO BIO’s PowerSoil DNA Isolation (Qiagen) kit.

### 4.6. Library Preparation and Illumina Sequencing

All DNA samples were subjected to bacterial 16S rRNA gene amplicon PCR. In the first round of 16S rRNA gene PCR, an amplicon of 444 bp was generated, using primers 341F and 785R [31], with an Illumina adapter overhang nucleotide sequence (underlined), resulting in the following sequences, 341F-adaptor: 5′-TCG TCG GCA GCG TCA GAT GTG TAT AAG AGA CAG CCT ACG GGN GGC WGC AG-3′ and 785R-adaptor: 5′-GTC TCG TGG GCT CGG AGA TGT GTA TAA GAG ACA GGA CTA CHV GGG TAT CTA ATC C-3′. Using the Q5 High-Fidelity DNA Polymerase system (M0491, NEB), a reaction volume of 25 μL per sample was prepared containing 1 μL of extracted DNA (final DNA-concentration per reaction 1–10 ng), 1× Q5 Reaction Buffer with 2 mM MgCl2, 200 μM dNTP mix, 1× Q5 High GC Enhancer (for the soil and fungi samples), 0.2 μM forward or reverse primer, and 1.2 U Q5 High-Fidelity DNA polymerase. For the seed endophytic samples, additionally 1 μL mitoPNA blocker (2 μM final concentration added from a 50 μM stock), 1 μL plastidPNA blocker (2 μM final concentration from 50 μM stock). The PCR program started with an initial denaturation for 3 min, at 98 °C, followed by a 30 s denaturation, at 98 °C, a 30 s annealing, at 57 °C, for V3V4 (58 °C for ITS) and a 1 min extension, at 72 °C, all three steps were repeated for a total of 35 cycles. The reaction was ended by a final 7 min extension, at 72 °C. The amplified DNA was purified using the AMPure XP beads (Beckman Coulter) and the MagMax magnetic particle processor (ThermoFisher, Leuven, Belgium). Subsequently, 5 μL of the cleaned PCR product was used for the second PCR attaching the Nextera indices (Nextera XT Index Kit v2 Set A (FC-131-2001), and D (FC-131-2004), Illumina, Belgium). For these PCR reactions, 5 μL of the purified PCR product was used in a 25 μL reaction volume and prepared following the 16S Metagenomic Sequencing Library Preparation Guide. PCR conditions were the same as described above, but the number of cycles reduced to 20, and 55 °C annealing temperature. PCR products were cleaned with the Agencourt AMPure XP kit, and then quantified using the Qubit dsDNA HS assay kit (Invitrogen) and the Qubit 2.0 Fluorometer (Invitrogen). Once the molarity of the sample was determined, the samples were diluted down to 4 nM using 10 mM Tris pH 8.5 prior to sequencing on the Illumina MiSeq. Samples were sequenced using the MiSeq Reagent Kit v3 (600 cycle) (MS-102-3003) and 15% PhiX Control v3 (FC-110-3001). For quality control, a DNA-extraction blank and PCR blank were included throughout the process, and the ZymoBIOMICS Microbial Mock Community Standard (D6300) to test efficiency of DNA extraction (Zymo Research).

### 4.7. Bioinformatic Processing of Reads

Sequences were demultiplexed using the Illumina Miseq software, and subsequently quality trimmed and primers removed using DADA2 1.10.1 [32] in R version 4.2.1. Parameters for length trimming were set to keep the first 290 bases of the forward read and 200 bases of the reverse read: maxN = 0, MaxEE = (2.5) and PhiX removal. Error rates were inferred, and the filtered reads were de-replicated and denoised using the DADA2 default parameters. After merging paired reads and removal of chimeras via the removeBimeraDenovo function, an amplicon sequence variant (ASV) table was built, and taxonomy assigned using the SILVA v138 training set [17]. The resulting ASVs and taxonomy tables were combined with the metadata file into a phyloseq object (Phyloseq, version 1.26.1) [33]. Raw reads are deposited in NCBI with SRA number: PRJNA916854.

### 4.8. Data Visualization and Statistical Analyses

The ASV table was further processed removing organelles (chloroplast, mitochondria), and prevalence filtered using a 2% inclusion threshold (unsupervised filtering) as described by Callahan et al., 2016 [32]. Alpha-diversity metrics such as observed ASV count, Simpson’s and Shannon’s diversity indexes were calculated on unfiltered data using scripts from the MicrobiomeSeq package. Hypothesis testing was performed using analysis of variance (ANOVA) and the Tukey Honest Significant Differences method (Tukey HSD). When assumptions of normality and homoscedasticity were not met, a Kruskal–Wallis Rank Sum test and a Wilcoxon Rank Sum test was performed. The results were summarized in boxplots. For beta-diversity, the Bray–Curtis, weighted and unweighted UniFrac distances were calculated on unfiltered data using the vegan package (version 2.5.4), and the data were visualized using a non-metric multidimensional scaling (NMDS). Relative abundances were calculated and visualized in bar charts using Phyloseq [33]. All graphs were generated in R version 4.2.1.

## 5. Conclusions

The seed endophytes of *N. caerulescens* are conserved regardless of location when compared at a population level, and therefore, we can expect all seeds to possess a similar internal microbiome. Isolates from the seeds reflected the community make up with the most predominantly culturable strains being from phylum proteobacteria. When isolates were applied to seeds of *A. thaliana*, growth was not significantly hindered or improved, except in one instance when root length was significantly improved (*p* < 0.01) when compared to the untreated control. Therefore, further study using isolated seed endophytes needs to consider the internal microbiome of the host seed, whether the introduction of foreign microbes to a conserved endophytic community is the way to increase plant mass and germination, and consider the presence of deleterious rhizobacteria found in weed species. We propose that possibly introducing microbes from phyla already present in the target seed is a potential way to move forward or through treatment of the seed with a consortium of bacteria to maximize the chance of success.

## Figures and Tables

**Table 1:** Identification of endophytic bacteria isolated from *N. caerulescens* seeds using sequencing results of the 16S rDNA gene Sanger sequencing. The identity percentage is the extent to which the given sequence aligns with the sequence found in the NCBI database.

Isolate nr	Closest NCBI Database Match	Identity (%)
1	*Mucilaginibacter* sp. 1042	94.81
2	*Methylobacterium* sp.	97.38
3	*Mucilaginibacter* sp.	94.78
4	*Noviherbaspirillum soli*	97.24
5	*Sphingomonas vulcanisoli*	95.46
6	*Sphingomonas* sp. AP4-R1	96.41
7	*Sphingomonas wittichii*	92.84
8	*Mesorhizobium ciceri*	97.85
9	*Paenibacillus* sp.	96.47
10	*Sphingomonas yunnanensis*	98.09
11	Alphaproteobact. (uncult.)	93.26

**Table 2:** Taxonomic classification of the isolated endophytic bacteria from *N. caerulescens* seeds, cultured on 869 media. The subdivision in ascending order of phylum, class, order, and genus was generated by the NCBI taxonomy browser.

Phyla	Class	Order	Genus
Proteobacteria	Alphaproteobacteria Betaproteobacteria	Rhizobiales Sphingomonadeles Burkholderiales	*Methylobacterium* sp. *Mesorhizobium ciceri* *Sphingomonas vulcanisoli* *Sphingomonas* sp. *AP4-R1* *Sphingomonas wittichii* *Sphingomonas yunnanensis* *Alphaproteobacteria bacterium* (uncultured) *Noviherbaspirillum soli*
Bacteroidetes	Sphingobacteriia	Sphingobacteriales	*Mucilaginibacter* sp. 1042 *Mucilaginibacter* sp.
Firmicutes	Bacilli	Bacillales	*Paenibacillus* sp.

**Table 3:** Physical and chemical characteristics of soil samples from Plombières and Overpelt, including bio-available trace elements, soil nutrient concentrations and total heavy metals (Zn, Cd and Pb). The heavy metal concentrations of Zinc (Zn), Cadmium (Cd) and Lead (Pb) are obtained from a previously conducted analysis.

	Plombières	Overpelt
Location	Plombières	Overpelt
	roadside, calamine area	tallud next to road
Point ID	waste tallud	waste tallud
Latitude	50.734	51.234
Longitude	5.964	5.386
Sampling date	18 June 2019	7 July 2019
Soil type	Organic/sand	organic
pH (KCl)	6.02	5.24
pH (H_2_O)	6.92	6.57
OC (% dry soil)	4.91	33.5
Conductivity (µS/cm)	129	108
NO_3_-N (mg/kg)	34	30
NH_4_-N (mg/kg)	3.4	7.2
N total (%/lds)	0.278	0.64
Fe (mg/100 g)	58.4	48.6
K (mg/100 g)	23	16
Mg (mg/100 g)	15	9.8
Ca (mg/100 g)	137	115
Mn (mg/100 g)	9.77	10.16
Na (mg/100 g)	<1.92	<1.92
*p* (mg/100 g)	11.4	6.6
C/N	17.7	52.4
K/Mg	1.5	1.6
Ca/Mg	9.1	11.7
Zinc (Zn) (mg/kg)	496	325
Cadmium (Cd) (mg/kg)	2.8	8.54
Lead (Pb) (mg/kg)	13,304	209

## Data Availability

Raw sequencing read were submitted to Short Read Archive (SRA, NCBI) and received the following BioProject ID: PRJNA916854.
